# Integrating Artificial Intelligence in Orthopedic Care: Advancements in Bone Care and Future Directions

**DOI:** 10.3390/bioengineering12050513

**Published:** 2025-05-13

**Authors:** Rahul Kumar, Kyle Sporn, Joshua Ong, Ethan Waisberg, Phani Paladugu, Swapna Vaja, Tamer Hage, Tejas C. Sekhar, Amar S. Vadhera, Alex Ngo, Nasif Zaman, Alireza Tavakkoli, Mouayad Masalkhi

**Affiliations:** 1 Department of Biochemistry and Molecular Biology, University of Miami Miller School of Medicine, 1600 NW 10th Ave, Miami, FL 33136, USA; axn668@miami.edu; 2 Rush Medical College, Chicago, IL 60612, USA; swapna_vaja@rush.edu (S.V.); tejas_sekhar@rush.edu (T.C.S.); 3 Norton College of Medicine, SUNY Upstate Medical University, Syracuse, NY 13210, USA; spornk@upstate.edu; 4 Kellogg Eye Center, Department of Ophthalmology and Visual Sciences, University of Michigan, Ann Arbor, MI 48105, USA; ongjo@med.umich.edu; 5 Department of Clinical Neurosciences, University of Cambridge, Downing Street, Cambridge CB2 3EH, UK; ew690@cam.ac.uk; 6 Sidney Kimmel Medical College, Thomas Jefferson University, 1025 Walnut St, Philadelphia, PA 19107, USA; phani.paladugu@students.jefferson.edu (P.P.); amarvadhera@gmail.com (A.S.V.); 7 Brigham and Women’s Hospital, Harvard Medical School, 75 Francis St, Boston, MA 02115, USA; 8 Department of Biological Sciences at Virginia Tech, Blacksburg, VA 24061, USA; tamerwsh@gmail.com; 9 Smith-Kettlewell Eye Research Institute, San Francisco, CA 94115, USA; zaman@nevada.unr.edu; 10 Human-Machine Perception Laboratory, Department of Computer Science and Engineering, University of Nevada, 1664 N Virginia St, Reno, NV 89557, USA; tavakkol@unr.edu; 11 School of Medicine, University College of Dublin, Belfield, D04 V1W8 Dublin, Ireland; mouayad.masalkhi1@gmail.com; 12 Department of Electronic & Computer Engineering, University of Limerick, V94 T9PX Limerick, Ireland

**Keywords:** orthopedic bioengineering, AI, interactive 3D models, diagnostic imaging, preoperative planning, smart implants, intraoperative robotics, bone regeneration devices, bone tumor management

## Abstract

Artificial intelligence (AI) is revolutionizing the field of orthopedic bioengineering by increasing diagnostic accuracy and surgical precision and improving patient outcomes. This review highlights using AI for orthopedics in preoperative planning, intraoperative robotics, smart implants, and bone regeneration. AI-powered imaging, automated 3D anatomical modeling, and robotic-assisted surgery have dramatically changed orthopedic practices. AI has improved surgical planning by enhancing complex image interpretation and providing augmented reality guidance to create highly accurate surgical strategies. Intraoperatively, robotic-assisted surgeries enhance accuracy and reduce human error while minimizing invasiveness. AI-powered smart implant sensors allow for in vivo monitoring, early complication detection, and individualized rehabilitation. It has also advanced bone regeneration devices and neuroprosthetics, highlighting its innovation capabilities. While AI advancements in orthopedics are exciting, challenges remain, like the need for standardized surgical system validation protocols, assessing ethical consequences of AI-derived decision-making, and using AI with bioprinting for tissue engineering. Future research should focus on proving the reliability and predictability of the performance of AI-pivoted systems and their adoption within clinical practice. This review synthesizes recent developments and highlights the increasing impact of AI in orthopedic bioengineering and its potential future effectiveness in bone care and beyond.

## 1. Introduction

Recent advancements in artificial intelligence (AI) are redefining orthopedic bioengineering, particularly in bone care, by enhancing precision, personalization, and postoperative outcomes [1,2]. These innovations, from AI-driven surgical planning to smart implants and bone regeneration technologies, are bridging gaps in traditional orthopedic practices while raising new ethical considerations [1,3]. This article synthesizes peer-reviewed findings and industry trends to explore AI’s transformative role in orthopedics.

**Figure 1:**
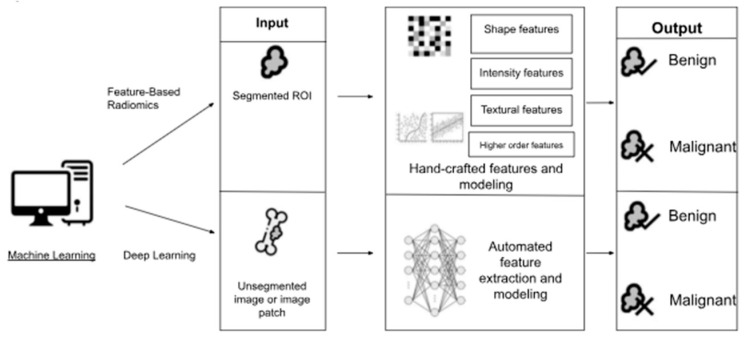
Diagram illustrating the differences between feature-based radiomics and deep learning, two types of machine learning. Adapted using Microsoft Word [5].

Incorporating AI into orthopedics has many applications, from improving preoperative protocols and intraoperative navigation to producing smart implants and hastening bone regeneration treatments [1,2]. AI’s effect in orthopedics is especially impactful for orthopedic diagnostics, as machine learning (ML) algorithms have been remarkably effective in assessing medical images (Figure 1) [4,5]. These developments improve diagnostic accuracy and allow for the earlier detection of bone tumors and orthopedic conditions, which can lead to improved patient outcomes [5,6].

A comprehensive review of 34 studies revealed that AI techniques achieved overall accuracy ranging from 0.44 to 0.99, sensitivity from 0.63 to 1.00, and specificity from 0.73 to 0.96 in distinguishing benign from malignant bone lesions across various imaging modalities [5]. For instance, researchers at the Foot & Ankle Research and Innovation Laboratory (FARIL) at Massachusetts General Hospital have created an innovative automated musculoskeletal image interpretation system (AMIIS) that improves diagnostic accuracy by more than 90% [4].

## 2. Methods

This review was conducted using a structured literature search of peer-reviewed publications from January 2018 to March 2025 across databases including PubMed, Scopus, and IEEE Xplore. The keywords included combinations of “artificial intelligence”, “machine learning”, “orthopedic surgery”, “bone regeneration”, “robotics”, “smart implants”, and “neuroprosthetics”. The inclusion criteria comprised English-language articles focused on the application of AI or machine learning in orthopedic bioengineering, including diagnostics, preoperative planning, surgical assistance, biomaterials, and rehabilitation technologies. The exclusion criteria included conference abstracts, editorials, non-English papers, and studies unrelated to orthopedic applications. After the initial screening of titles and abstracts, 59 studies were selected for full-text analysis and thematic synthesis. Priority was given to studies reporting performance metrics, clinical outcomes, or technological innovation in AI-assisted orthopedic care.

## 3. Discussion

### 3.1. AI’s Transformative Role in Orthopedic Bioengineering

**Figure 2:**
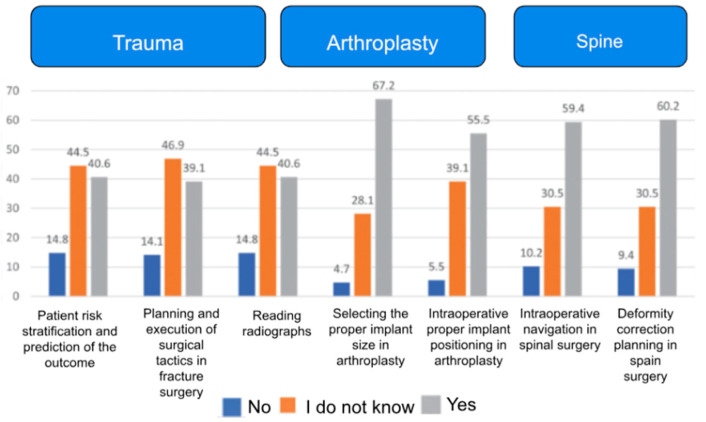
Bar chart showing surgeons’ responses to questions about AI applications in orthopedics (N = 128). Adapted using Microsoft Word [7].

**Figure 3:**
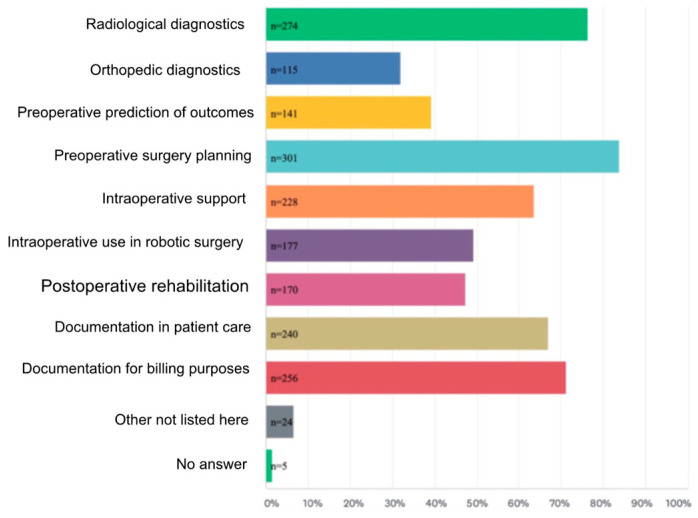
Anticipated clinical applications of artificial intelligence in orthopedics, based on multiple-choice responses. Bar lengths represent the frequency at which each application was selected by respondents. Preoperative surgical planning was the most highly anticipated application 83.8%. Adapted using Microsoft Word [8].

AI’s role in orthopedic bioengineering, particularly in joint reconstruction, spine surgery, and trauma care, has been extensively reviewed. Studies highlight its ability to enhance precision and personalization in surgical outcomes while addressing challenges like ethical considerations and regulatory gaps (Figure 2 and Figure 3) [1,3,7]. Additionally, Rupp et al. 2024 [8], conducted an online, cross-sectional survey on 360 orthopedic surgeons of the AGA Society for Arthroscopy and Joint Surgery. They found that 54.5% of them expected AI to complement the field of orthopedics within the next decade, with preoperative planning being the most likely clinical use (83.8%) [8].

**Figure 4:**
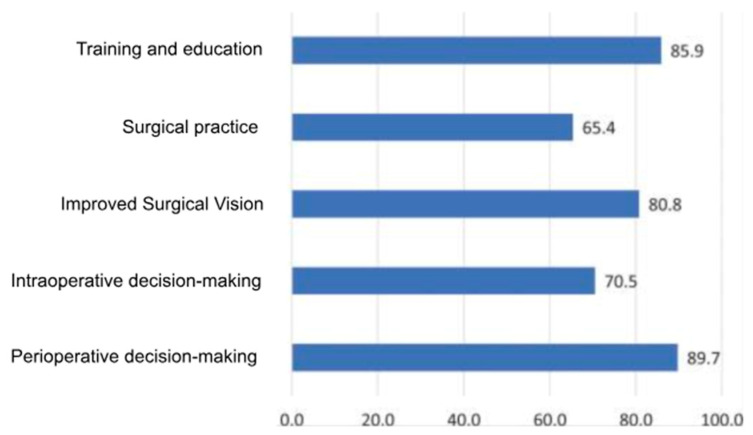
Bar chart showing percentage of surgeons’ responses to the usefulness of AI in orthopedics (N = 128) [7].

AI-driven innovations, such as 3D anatomical modeling and surgical planning (Figure 2 and Figure 3), are bridging gaps in traditional practices by improving diagnostic accuracy and enabling patient-specific interventions [9,10,11], as further supported by survey data (Figure 4) showing strong surgeon endorsement of AI’s clinical usefulness in orthopedics.

### 3.2. AI in Preoperative Planning and Surgical Optimization

**Figure 5:**
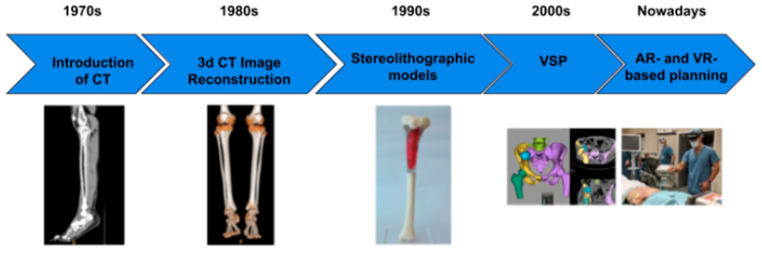
A timeline of the development of visualization techniques used for presurgical planning in orthopedic settings. Adapted from Microsoft Word [11].

**Figure 6:**
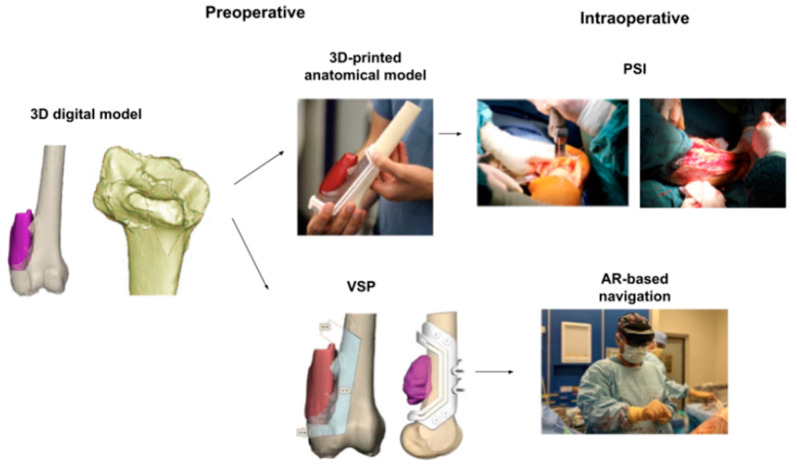
Applications that used combined features of 3D technologies. Adapted from Microsoft Word. Permission obtained from Wikimedia Commons: This file is made available under the Creative Commons CC0 1.0 Universal Public Domain Dedication [11,13,14,15].

**Figure 7:**
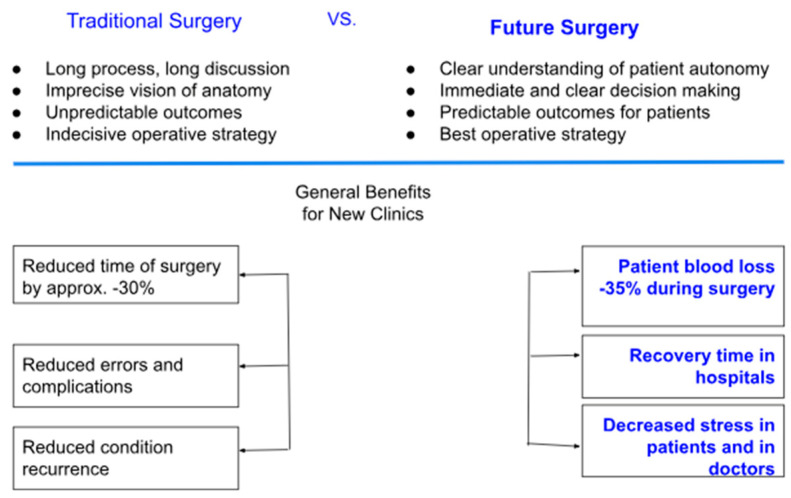
Comparative analysis showing AI’s benefits in orthopedic surgeries in the pre-, peri-, and postoperative settings. Adapted from Microsft Word [10].

AI is revolutionizing preoperative workflows by automating the creation of 3D anatomical models. Traditional manual segmentation of CT/MRI scans, which normally takes weeks to complete, is now accomplished in minutes using AI algorithms (Figure 5), reducing radiation exposure by enabling X-ray-based 3D reconstructions, reducing manual segmentation time, and improving preoperative planning (Figure 6) [1]. For instance, AI-powered tools like Enhatch provide surgeons with real-time, patient-specific models to optimize implant alignment and predict surgical outcomes (Figure 6 and Figure 7). Such precision is critical in joint replacements, where even millimeter-level deviations impact long-term functionality [9,10,12].

These AI-driven systems reduce radiation exposure by enabling X-ray-based 3D reconstructions, as seen in platforms like TiRobot, which cuts intraoperative X-ray use by 70% [1,11]. Beyond planning, AI’s integration in orthopedic surgery has led to measurable clinical improvements, including a 30% reduction in operative time, a 35% decrease in patient blood loss, fewer surgical complications, and faster hospital recovery times (Figure 7).

Beyond model generation, AI plays an increasingly critical role in stereotactic navigation and intraoperative accuracy. Systems like TiRobot integrate AI-assisted stereotactic guidance to achieve sub-millimeter precision in implant positioning by leveraging real-time imaging data with preoperative models. These systems enhance targeting accuracy and reduce variability in complex procedures, especially in spinal and pelvic surgeries [11,16,17]. Complementing these systems are AI-driven imaging tools—particularly those powered by convolutional neural networks (CNNs)—which automatically identify and segment regions of interest (ROIs) within CT and MRI scans. This facilitates enhanced visualization of critical anatomical structures, guiding implant placement with exceptional precision [17,18,19,20]. ROI-based planning not only improves alignment but also shortens operative time and minimizes the risk of iatrogenic injury through personalized, data-driven trajectory optimization [1,2,21]. Moreover, CNNs have demonstrated >95% sensitivity in detecting subtle bone defects and grading osteoarthritis, comparable to expert radiologists [1,22]. Another 2024 clinical study on TiRobot demonstrated a 20% increase in surgical efficiency and a 70% reduction in radiation exposure during complex spinal and trauma surgeries [11].

While not femoral-specific, this aligns with AI’s role in optimizing fixation strategies through real-time anatomical tracking and preoperative 3D planning. Similarly, Stryker’s Mako system uses CT-derived 3D modeling to optimize implant size and alignment with sub-millimeter precision, minimizing intraoperative errors and improving long-term functionality in joint replacements [7,10]. Kolomenskaya et al. (2023) demonstrated that AI models analyzing biomechanical data and patient-specific factors can recommend tailored fixation strategies, reducing postoperative complications by 32% compared to traditional methods [1,7,23].

### 3.3. AI in Bone Grafting and Biomaterial Innovation

AI also transforms bone grafting and implant material selection, enhancing product durability and patient outcomes. Traditional bone grafting methods, such as autogenic grafts, are constrained by limited donor tissue availability and tissue harvesting complications. Likewise, allogeneic grafts carry risks of immune rejection and pathogen transmission. These challenges have driven interest in synthetic and composite materials for bone repair [23].

AI is accelerating biomaterial innovation by optimizing their composition and structural properties. Bioceramics, such as hydroxyapatite (HAp)-based materials, offer superior biocompatibility, osteoconductivity, and bioactivity, making them viable alternatives to traditional grafts. AI-driven models streamline biomaterial selection by analyzing crucial physiologic factors like porosity, degradation rates, and mechanical properties to enhance bone integration [23,24].

**Figure 8:**
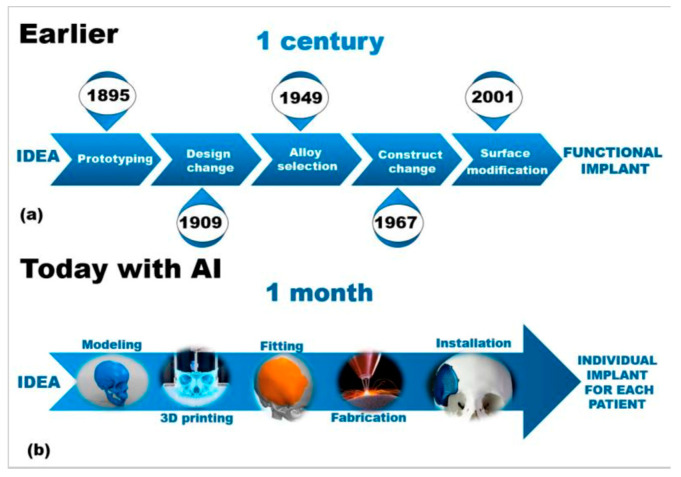
Sample representation of a medical engineering workflow, comparing the traditional, century-long implant development process (**a**) with the modern AI-assisted approach enabling patient-specific implants within one month (**b**) [1,7,23].

**Figure 9:**
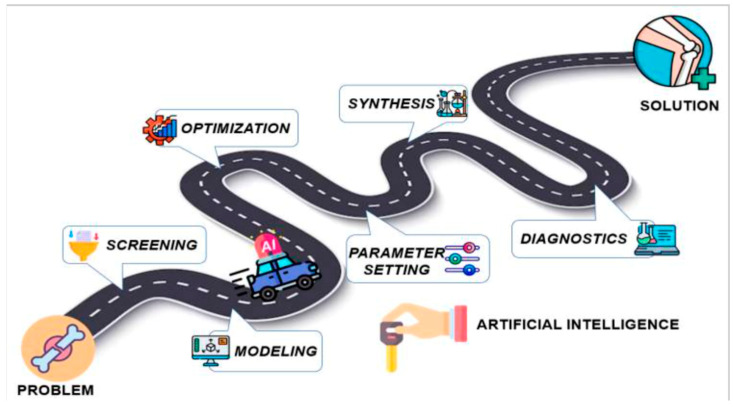
Sample pathway for integration of AI into medicinal chemistry to accelerate the design of novel materials in tissue engineering [1,7,23].

Moreover, AI has changed scaffold fabrication for bone tissue engineering tremendously. Instead of the previously existing, often slow and labor-intensive trial-and-error procedures, AI-enabled approaches to scaffold design enable the prediction of material behavior and assist in refining structures appropriately (Figure 8). With some materials (e.g., bioactive glasses) having controlled release of ions to stimulate osteogenesis and vascularization, AI-enabled techniques, like 3D printing and electrospinning, facilitate the creation of customized scaffolds with optimized porosity and drug delivery [25]. Composite scaffolds are useful for incorporating organic and inorganic materials to enhance mechanical integrity and bioactivity [25]. These AI-enabled scaffold improvements in orthopedics have led to better patient outcomes using shorter surgical times and fewer complications (Figure 9) [10].

Additionally, by accelerating project timelines, AI-enabled scaffold improvements can greatly reduce the time spent on research and development from initial proof of concept to patient use [26]. Despite the rapid improvements, there are still challenges in ensuring ethical and regulatory approval for AI-generated materials. Future research should focus on improving AI models for clinical applications related to parameters such as longevity of the material in the body and biocompatibility. Incorporating AI into biomaterials in addition to surgical options is a significant step towards disrupting current orthopedic medicine and creating more efficacious orthopedic treatment options, as well as individualized treatment approaches [26].

### 3.4. Machine Learning and Neural Networks in Implantology

Earlier AI models in medicine often relied on logic-based and symbolic methods, which lacked the precision and predictive power of modern ML and deep learning (DL) algorithms. ML allows systems to learn from data without explicit programming, using techniques such as Bayesian networks, ensemble methods, and gradient boosting to improve diagnostics and treatment planning, such as predicting dental and orthopedic implant needs [25,26].

DL, a subset of ML, employs artificial neural networks (ANNs) with multiple layers, enhancing the ability to recognize complex patterns and solve sophisticated tasks. ANNs, inspired by the structure of the human brain, process information through interconnected nodes that adaptively learn from data. In implantology, ANN models help identify subtle factors influencing implant success, predict long-term viability, and reduce complications at all stages of treatment [25,26].

For example, a neural network trained on data from over 1600 patients achieved a 94.48% accuracy rate in predicting implant survival using ReLU and softmax activation functions [26]. The model analyzed 55 statistical factors through one-hot encoding to classify implants as either “survival” or “rejection” [26]. These AI-driven innovations underscore the potential of intelligent algorithms in revolutionizing orthopedic bioengineering, paving the way for safer, more effective, and highly customized patient care [25,26].

While ANNs have been foundational, other ML models continue to gain traction in biomedical and orthopedic applications. Random Forests are frequently used in classification tasks because of their resilience to overfitting and ability to manage complex datasets. For example, Random Forests have been shown to effectively predict clinical outcomes, such as moderate to severe acute postoperative pain after orthopedic surgery, outperforming traditional logistic regression models in terms of classification error and area under the curve (AUC) [27].

Support Vector Machines (SVMs) are applied to biomechanical modeling, including tasks like distinguishing implant types and predicting mechanical failure under stress. SVMs are valued for their robustness in handling high-dimensional data and their effectiveness in classification scenarios, as demonstrated in various machine learning tutorials and practical applications using scikit-learn and related frameworks [28]. Bayesian networks have informed clinical risk stratification tools by integrating clinical and non-clinical factors to model the probability of outcomes, such as time to return to sport and injury severity. These networks can support individualized clinical decision-making and scenario planning, as shown in recent studies involving professional athletes [29].

Reinforcement learning algorithms are being introduced in adaptive systems that optimize implant placement by responding to real-time biomechanical feedback. While peer-reviewed clinical studies on reinforcement learning in orthopedic implantology are still emerging, the potential for adaptive, data-driven decision support is recognized in the field. These diverse models are typically developed using powerful open-source frameworks, such as TensorFlow, PyTorch, and scikit-learn, which provide accessible APIs and support a wide range of machine learning techniques. AutoML platforms, including Auto-Sklearn and Auto-PyTorch, further streamline the process by automating model selection and hyperparameter tuning, allowing for efficient development and deployment of machine learning models in clinical and research settings [28]. Together, this ecosystem of ML tools is driving a new era of precision and personalization in orthopedic implantology, enabling more accurate predictions, individualized treatment planning, and improved patient outcomes [27,28,29].

### 3.5. AI in Bone Tumor Diagnosis and Treatment

AI is also advancing the diagnosis, treatment, and management of various bone tumors in orthopedics, specifically in differentiating between benign (osteoid osteoma and osteochondroma) and malignant tumors (osteosarcoma and chondrosarcoma).

AI has shown great potential to differentiate between benign and malignant bone lesions in radiological reviews across multiple imaging modalities [30]. In a systematic review of 34 studies, AI approaches demonstrated overall accuracy from 0.44 to 0.99, sensitivity from 0.63 to 1.00, and specificity from 0.73 to 0.96 for distinguishing benign from malignant bone lesions [30]. These AI models have been successfully applied in radiographs, MRI, CT, and PET/CT scans, displaying various applications in medical image analysis. Furthermore, in CT-based radiomics, AI models could differentiate atypical cartilaginous tumors from high-grade chondrosarcoma with accuracy that was either on par or superior to preoperative biopsy results [30].

Beyond imaging, ML models now assess tumor pathology at a cellular level, offering more accurate prognoses than conventional methods. For osteosarcoma—the most common malignant bone tumor—a machine learning model developed at Kyushu University evaluates the density of surviving tumor cells after treatment [30]. This model matches pathologist assessments while offering improved consistency and efficiency in predicting tumor response to therapy.

Furthermore, AI-assisted tumor pathology analysis eliminates human variability in identifying residual viable tumor cells, allowing for more precise treatment response assessment and personalized therapeutic planning.

### 3.6. Intraoperative Robotics and Precision Surgery

Robotic-assisted systems are now standard in total knee arthroplasty (TKA) and spinal surgeries, with the global orthopedic robotics market projected to reach USD 16 billion by 2030 [9]. Platforms like Stryker’s Mako system leverage AI to execute pre-mapped surgical plans with sub-millimeter accuracy, minimizing soft-tissue damage and improving implant longevity [3,31]. Real-time AI feedback adjusts for intraoperative anatomical shifts, enhancing reproducibility in osteotomies and joint reconstructions [3]. During knee and hip replacements, these systems ensure precise bone cuts, optimal alignment of prosthetic joints, and minimal soft tissue damage, leading to better patient outcomes, faster recovery times, and fewer postoperative complications (Table 1) [31].

Over the past two decades, robotic technology in orthopedics has evolved from passive systems, where surgeons retain full control, to active systems, where robots autonomously perform tasks. Semiactive or “haptic technology”—as seen in robotic-arm-assisted TKA—strikes a balance by providing real-time feedback, ensuring precise bone resection and soft-tissue balancing. Robotic systems like Mako and ROSA Knee leverage AI to execute pre-mapped surgical plans with <1° deviation in implant alignment, further improving surgical precision [10,32].

AI-driven advancements in robotic platforms have significantly improved surgical outcomes (Table 2). For instance, Mako’s semi-autonomous robotic arm achieves 99.9% accuracy in hip–knee angulation within ±3° of planned targets, enhancing implant positioning and alignment. Similarly, OMNI-Botics utilizes AI-driven tensioning sensors to balance ligaments intraoperatively, leading to a 99.48% implant survival rate at a 6-year follow-up [32]. Another breakthrough, TiRobot, reduces operative time by 20% through automated instrument positioning and real-time optical tracking, optimizing efficiency and precision in orthopedic procedures [10,32].

### 3.7. Smart Implants and Remote Monitoring

Smart implants integrated with biosensors facilitate proactive postoperative care by allowing continuous real-time monitoring of joint function outside the clinical environment [9,23]. For example, Zimmer Biomet’s Persona IQ knee implant monitors gait pattern, step count, and load distribution to transmit data to clinicians, which allows them to identify complications (e.g., loosening, infection, or abnormal biomechanics) before clinical symptoms arise [9,23]. Studies have found that sensor monitoring allowed for early intervention in 32% of patients who underwent TKA, effectively reducing the revision rate and improving long-term outcomes [10].

Integrating AI-based predictive models expands the functionality of smart implants. AI can triangulate sensor data with patient biomarkers (e.g., inflammatory cytokines) to predict the risk of implant failure before clinical symptoms arise, paving the pathway for personalized and preemptive intervention. In addition, autonomous or AI-enabled robotic systems used in orthopedic surgeries can utilize real-time feedback for optimal implant positioning and alignment, with sub-millimeter accuracy in total knee and hip arthroplasty, amongst other procedures. This data-driven strategy can reduce postoperative complications, improve rehabilitation times, and extend the lifespan of the implant [26].

Furthermore, advances in materials science and additive manufacturing emphasize the potential of smart implant technology. Mass customization of orthopedic implants can be realized through 3D printing and robotics to ensure a patient-specific fit while maintaining optimal biomechanical properties [25]. Titanium alloys and bioactive polymers improve osseointegration, in addition to drug-releasing polymer frameworks, which can help moderate inflammatory reactions [24]. Electrospinning is an example of a technique used to create cell-adhered scaffolds that aid bone regrowth and provide stability to the implant [25].

Nonsurgical protocols are also critical design components for implant design and performance prediction. Finite element analysis (FEA) and graph-based modeling can assess the bone–implant interface, studying parameters such as friction coefficient, porosity, and load distribution [26]. These models create better biomimetic implants that replicate some of the properties of actual bone and can minimize or eliminate mechanical mismatch and long-term durability issues [23].

As smart implants evolve, their integration with AI, biosensors, and regenerative materials represents a paradigm shift toward proactive, data-driven orthopedic care. With real-time remote monitoring, predictive analytics, and personalized interventions, the future of implantable electronics is poised to significantly improve patient outcomes and surgical success rates [26].

### 3.8. AI-Driven Bone Regeneration and Neuroprosthetics

AI is transforming neuroprosthetics and bone regeneration by significantly improving the precision, adaptability, and effectiveness of medical care (Table 3). Orthopedics and neuroprosthetics have historically provided vital interventions for bone injuries and motor impairments, but they suffer from important limitations, such as incomplete restoration of function and slow recovery. AI advances neuroprosthetics that respond to key limitations by offering more adaptive and personalized solutions. For neuroprosthetics, AI enhances communication between the brain and affected limb using adaptive prostheses, real-time adaptive control systems, and brain–computer interfaces (BCIs) [33,34]. This allows neuroprosthetics to learn and adapt to the patient’s movement patterns, providing greater control and more natural function. Össur and ReWalk Robotics are two companies using AI to develop adaptive prosthetic devices, like Össur’s Proprio system and the ReWalk exoskeleton that adjust automatically to ground conditions or read brain signals to achieve more natural arms, legs, or torso movements [34].

In the realm of bone regeneration, AI is driving advancements in both surgical planning and material design (Table 4). Companies like Bioventus and Xtant Medical are integrating AI into their bone regeneration solutions, using predictive modeling to improve implant designs and accelerate healing. AI-enhanced 3D printing and generative design algorithms optimize scaffold structure for bone repair, balancing porosity and load distribution to enhance osseointegration and reduce recovery time. For instance, machine learning techniques are used to design bioceramic scaffolds that promote faster bone healing, with trials showing up to 40% faster healing times than traditional methods. These AI-driven innovations are particularly beneficial in complex fractures, osteoporosis, and bone tumors, where tailored implants and biologically active materials speed up healing and reduce complications.

Moreover, AI is enabling breakthroughs in neuroprosthetics for patients with spinal cord injuries, stroke, ALS, and cerebral palsy by improving the functionality of adaptive prostheses and BCIs (Table 5). By decoding neural signals, AI helps control prosthetics more intuitively, restoring motor function and enhancing mobility for patients with severe impairments. For bone regeneration, AI improves treatments for complex fractures, advanced osteoporosis, and traumatic injuries by customizing scaffold designs to optimize bone repair and reduce recovery times [33,34].

Pioneering companies such as Neuralink and Medtronic are actively working on cutting-edge BCIs that incorporate advanced AI and are intended to restore motor function. Meanwhile, companies such as RevBio and Bioretec use AI to develop new therapies that support bone regeneration using bioactive ceramics and 3D-printed implants. These two approaches are fundamentally transforming how we think about treatment for healing damaged bone and recovering lost motor function, ultimately offering patients more effective, patient-specific, and less invasive approaches to treatment (Table 6).

By combining AI-driven approaches in neuroprosthetics and bone regeneration, we are witnessing a paradigm shift in medical technology that promises to restore function and mobility and significantly improve the quality of life for patients facing serious medical challenges.

## 4. Ethical and Practical Considerations

**Figure 10:**
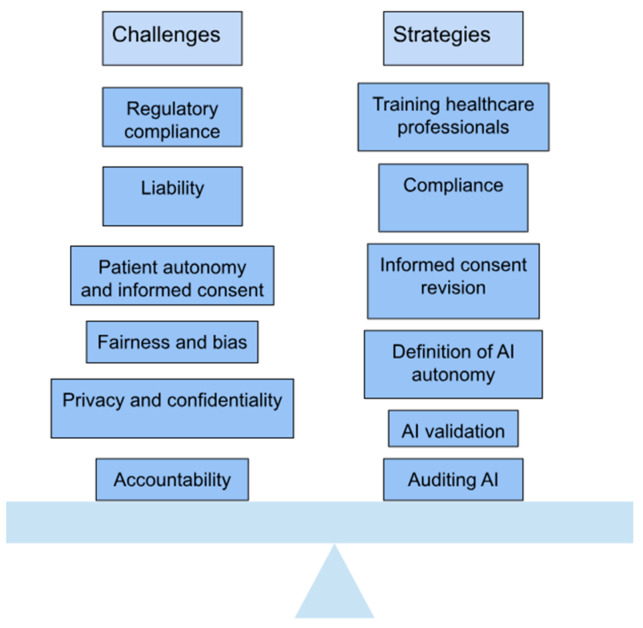
Balancing challenges and strategies for implementing AI in healthcare. Adapted from Microsoft Word [76].

The increasing use of AI in orthopedic bioengineering has led to remarkable advances in patient care and surgical accuracy. Still, it presents several ethical and practical challenges that must be addressed carefully (Figure 10). A particular concern is data bias, as AI algorithms often utilize datasets that do not necessarily represent the diversity in the population. Without accounting for diversity, AI may introduce inaccuracies into the evaluative process, particularly for underrepresented patients. For instance, AI models achieve their optimal performance through training data collected largely from patients of European or North American descent. Yet, they may not reflect the anatomical differences found among other patient groups, making their outputs less effective for the latter patients. This example illustrates why it is critical to employ diverse datasets when training AI models so that they exhibit equitable performance across all patient populations [3,26,75].

Along with data bias, there is also concern about manual surgical skills being erased due to the increasing use of AI. For example, a 2023 study showed that residents trained solely by robotic-assisted systems performed TKA many times less accurately than residents trained by open techniques. This phenomenon is generally referred to as deskilling, and it represents a major risk, particularly when the surgeon may not be able to resolve a failed artificial intelligence system or if the system malfunctions in the operating room. In these situations, a complete system collapse may result from an active threat, malware challenge, or technical error. For these reasons, it is important that trainees also practice conventional methods even while building their expertise in AI-assisted surgery [3,26,77].

Regulatory gaps also complicate the adoption of AI in orthopedics. Although AI may increase precision and decrease human errors, many AI-assisted orthopedic devices are not fully tested in real-world applications. The FDA found that only 50% of devices underwent dynamic clinical validation. Additionally, clinical testing often occurs within controlled environments, hindering the validation of devices in real clinical practice with variable clinical scenarios. Furthermore, suppose an AI-assisted device has not been fully tested in a clinical environment. In that case, it questions the reliability and validity of the device when employed outside of a controlled laboratory, necessitating standardized regulatory guidelines to validate AI systems prior to implementation in clinical practice [3,26,78].

Finally, introducing AI systems into medicine must align with established ethical principles in using AI technologies. For example, the Declaration of Geneva of the World Medical Association states that AI technologies should not discriminate based on age, gender, race, or other social factors. Addressing overt discrimination in an AI system is relatively straightforward; however, biases that result from unconscious bias in the training datasets are more problematic. AI systems must be made safe from bias, and a dataset must be monitored and continuously updated to reflect the general patient cohort and continuously evolve given novel medical practice [3,75,79]. The ethical implications of a patient’s privacy and consent remain significant issues as AI-driven systems reduce engagement. AI systems do not afford the patient a true dialogue, as AI systems do not truly engage or offer any consenting process [3,77].

The concept of informed consent becomes increasingly complicated when AI systems act like “black boxes”, so that neither the patient nor the physician thoroughly comprehends the underlying decision-making process. In these instances, it is technically possible for patients to give consent, but they do not truly understand what consent means in terms of procedures that the AI supports, so the consent becomes meaningless [75,77,78,79,80]. Likewise, the use of AI for surgical procedures, like a robot making an incision or guiding the procedure in TKA, creates situations that may lead to excessive deference to technology, adversely affecting the surgeon’s confidence and skill when using this potentially faulty technology. This problem may also lead to significant concerns if AI algorithms make faulty recommendations because surgeons rely on AI without questioning the recommended intervention [80,81].

Another important issue is cybersecurity. As AI technologies in orthopedics become more mainstream, the likelihood of cyberattacks against AI-enabled systems will increase. A breach involving the theft of data or compromised software systems could have severe implications, including providing incorrect surgical guidance or causing data breaches. A study published in HealthITAnalytics (2024) indicates that black box AI tools often lack adequate cybersecurity controls, making them amenable to attacks that could compromise patient safety and data integrity [82]. This points to an important need for strong cybersecurity controls to protect patient data and ensure the integrity of AI systems [75,77,78,79,80,81,82].

**Figure 11:**
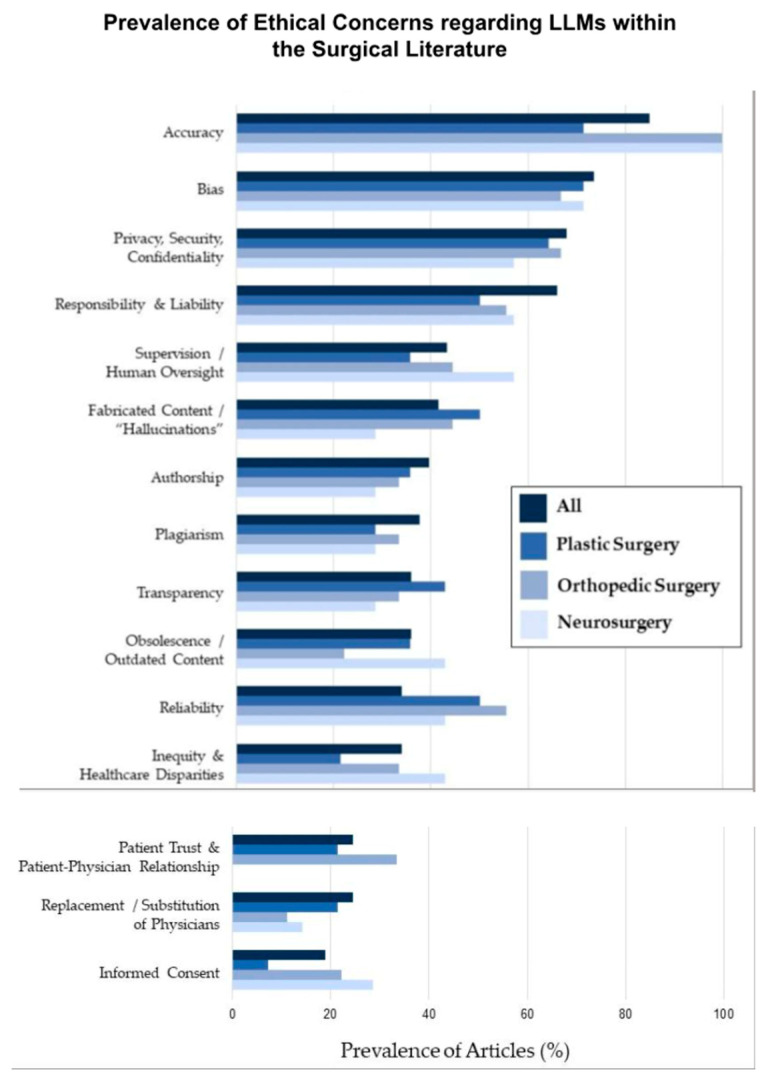
Graphical representation of the ethical concerns regarding LLMs within several surgical practices, as well as their prevalence [77].

Lastly, accountability for using AI technologies raises the next significant ethical issue. When an AI system fails or has an adverse outcome, it is unclear whether accountability lies with the software developer, the healthcare provider, or the regulatory agency that sanctioned the technology. This uncertainty complicates the ethical and legal framework around the use of AI in medicine. Research published in BMJ Medical Ethics (2021) points to the alarming ethical questions arising from black box algorithms since there is a lack of accountability and transparency when adverse events occur through the algorithm’s opaque decision-making process [81,82]. Addressing these concerns requires a joint effort among engineers, clinicians, and policymakers to produce a structure for developing standards of ethical use of AI (Figure 11).

As AI-driven innovations evolve, they offer enormous potential to transform orthopedic practices, but only if these ethical and practical challenges are carefully managed. By addressing issues such as data bias, surgeon deskilling, regulatory gaps, and accountability, the healthcare industry can maximize the benefits of AI while minimizing its risks [76].

## 5. Future Directions

The future of orthopedics appears set for significant advances, powered by accelerating developments in AI, biomaterials, and nascent technologies, such as 3D bioprinting. AI is revolutionizing how patients are cared for because of improved accuracy of diagnostics and enhancements in surgical planning, risk assessment, and postoperative recovery monitoring. For example, using an AI tool that generates 3D anatomical models from imaging data takes only a few clicks. It can dramatically reduce the time it takes to set up a preop plan and improve the procedure’s accuracy (e.g., knee replacement, fracture repair) [9,83]. AI algorithms can also analyze large datasets, recommend individualized surgical approaches, predict outcomes, and provide real-time feedback to surgeons [84].

Notwithstanding the insights of AI, challenges remain, particularly regarding regulatory frameworks and addressing ethical issues, including the privacy of data and algorithmic bias, which remain barriers to implementation. Countries have begun to put in place regulatory standards to optimize the clinical use of AI. Still, it will remain critical to continue to develop guidelines on dataset curation and algorithmic transparency of AI to ensure ethical use [76,82,83,84].

Regarding materials, the advancements in orthopedic implants are redefining practices with improved durability and a more favorable experience for patients. Utilizing 3D-printed titanium alloys, highly cross-linked polyethylene, and bioresorbable polymers enhances longevity and biocompatibility in implants with reduced infection rates. The Persona^®^ Solution™ PPS^®^ Femur from Zimmer Biomet has an innovative porous design that provides novel options for patients sensitive to traditional materials. At the same time, Onkos Surgical has produced an FDA-approved antibacterial covering for their tumor and revision implants that invokes a modern approach to infection prevention with antibiotics in implant care. Bioresorbable implants, like screws and plates, are gaining traction, with a projected market of USD 16.35 billion by 2035 [85,86]. These materials become resorbed over time, eliminating follow-up surgeries for hardware removal [86].

Integrating AI and 3D bioprinting will help expedite orthopedic bioengineering therapy by creating patient-specific bone grafts based on individual anatomy to decrease the mismatch between patient anatomy and implant materials, improving surgical outcomes. For example, smart implants with embedded sensors can remotely access data on healing rates to measure a limb’s range of motion and alignment without an invasive procedure [83,84]. Large-scale clinical trials are needed across multiple populations to verify these procedures and innovations further. The ROSA Knee system provided a 99.26% survival rate at 3 years post-implantation in a study containing a high number (766) of patients, indicating the possibility of smart implants to significantly evolve the overall quality of care in the orthopedic setting [9,83,84].

The orthopedic future is moving towards robotics and advanced materials to deliver this type of care into everyday clinical practice in the near term. The utilization of robotics and advanced materials will yield further enhanced patient outcomes and more accessible orthopedic care due to the different and varied setups that robotics entails. In this evolving landscape, orthopedic care will only further evolve into the patient’s needs and centered access to individualized care. The overall future of orthopedics is one of the exciting possibilities in re-establishing a field we sometimes do not think of as “scientific”. It unifies the newest possibilities of robotics and advanced materials while still holding to the ethical responsibilities of our profession, dedicated to improving the quality of life for our patients across the world and, maybe even one day, to achieving a proposed global equitable healthcare system.

## 6. Conclusions

AI integration in orthopedic bioengineering has led to a definitive change in patient management with increased precision, personalization, and efficiency. These innovations are changing the face of orthopedic practice, from AI-assisted diagnostic tools to smart implants and regenerative therapies. For example, AI algorithms have been shown to have very high sensitivity (94%) and specificity (91%) when detecting osteoarthritis at accuracies comparable to fellowship-trained arthroplasty surgeons [87]. ML models have even been developed to optimize the preoperative plan for procedures such as total knee arthroplasty (TKA), predicting component size with accuracy from 88.3% to 99.9% [87]. However, challenges remain, including data bias, automated reliance on AI systems, and regulatory limitations. Ethical issues, such as algorithm transparency, patient privacy, and accountability of adverse events, must also be considered [4].

Multidisciplinary collaboration will be crucial as the field evolves to address these challenges and ensure ethical AI deployment. Future research should focus on large-scale clinical trials to validate AI technologies and their impact on patient outcomes. For instance, studies have shown that robotic-assisted surgeries improve implant alignment and soft tissue balance in TKA, but further research is needed to assess reproducibility across diverse clinical settings [1,3]. Developing standardized protocols for algorithm transparency is another critical step. Transparent AI systems can help build trust among clinicians by highlighting key radiologic features used in decision-making processes, as demonstrated by deep learning models for diagnosing osteoarthritis [1]. Additionally, exploring novel applications such as AI-driven bioprinting could revolutionize the creation of patient-specific implants and prosthetics by tailoring designs based on anatomical data [88].

By embracing these innovations responsibly, the orthopedic community can harness AI’s potential to significantly improve patient outcomes and advance the field of bone care. For example, generative AI has shown promise in designing customized implants that enhance comfort and functionality by analyzing parameters like bone density and movement patterns [88]. As we move forward, continued research and development in AI applications will undoubtedly play a pivotal role in shaping the future of orthopedic bioengineering. This progress promises more precise, accessible, and personalized treatment options for patients worldwide.

## Figures and Tables

**Table 1:** Traditional surgery vs. AI-driven surgery.

Traditional Surgery	Robotic-Assisted Surgery
Manual implant alignment	Algorithm-driven precision
Higher risk of human error	Reduced variability (<1° deviation)
Extended recovery periods	Faster mobilization (e.g., 20% shorter hospital stays)

**Table 2:** Various metrics showing the improvement in AI–robotic TKA over traditional TKA.

Metric	Traditional TKA	AI–Robotic TKA
Implant alignment accuracy	69.9% within target	99.9% within target
Postoperative ROM accuracy	Slower	20% faster recovery
Radiation exposure	High	Reduced by 70%

**Table 3:** Leading AI companies in neuroprostheses.

Company	Focus	AI Application
Össur [34,35,36]	Advanced neuroprosthetics and orthopedic technologies	Employs AI to improve the functionality of prosthetics, exemplified by the Proprio knee prosthesis, which dynamically adapts to terrain and walking speed.
ReWalk Robotics [34,37,38]	AI-developed exoskeletons for individuals with mobility impairments	Utilizes AI to interpret user intent and modulate exoskeleton movements, enabling more natural and responsive walking patterns.
Neuralink [34,39]	Brain–computer interface (BCIs) development for neurological disorders	Designs AI-driven BCIs for direct brain-to-device communication, targeting functional restoration in individuals with paralysis.
Medtronic [34,40,41,42]	Neurosurgical medical devices and surgical systems	Integrates AI to enhance precision in device implantation and tailor interventions for neurological injuries and diseases
Bionik Laboratories [34,43,44]	Rehabilitation technologies and neuroprosthetics	Develops AI-powered exoskeletons that respond adaptively to user movement, promoting mobility recovery for patients with paralysis.
NeuroPace [34,45,46,47]	Implantable neurostimulation devices	Uses AI to customize and adjust brain stimulation therapies in real time, optimizing treatment for individual patients.
BrainCo [34,48]	Cognitive and motor neurotechnology solutions	Leverages AI to support the development of neuroprosthetics and enhance brain function and control.

**Table 4:** Leading AI companies in bone regeneration.

Company	Focus	AI Application
Xtant Medical [34,49,50]	Bone regeneration through surgical interventions	Applies AI in medical devices to improve implant integration and speed up the bone healing process
Bioventus [34,51,52,53,54,55,56]	Bone healing via stimulation technologies and cell therapies	Employs advanced AI to tailor therapies and enhance recovery for fractures and skeletal conditions
Orthofix [34,57,58]	Treatments for musculoskeletal disorders and bone repair	Integrates AI with electronic bone stimulation to support regeneration following surgical procedures
RevBio [34,59,60]	Biologically based therapies for bone repair	Uses AI to develop engineered biomaterials that facilitate healing in complex bone fractures
Bioretec [34,61,62,63]	AI-driven solutions for bone regeneration	Applies AI to design and optimize healing devices tailored to fracture-specific structural requirements

**Table 5:** Health challenges in neuroprostheses that AI can resolve.

Health Challenge	Technology	Surgical Technique	Benefit
Spinal Cord Injuries (SCIs) [34,39,40,41,42,64]	Brain–computer interfaces (BCIs) by Neuralink, adaptive exoskeletons from ReWalk Robotics and Ekso Bionics	Exoskeletons require no surgery (external devices); BCIs involve surgical implantation of brain electrodes	Restoration of partial or full mobility, increased independence, and improved quality of life
Stroke (CVA) [34,43,44,65]	Smart prosthetics with adaptive control systems by Bionik Laboratories	Minimally invasive procedures for implanting sensors and electrodes in neuroprosthetics	Enhanced rehabilitation through prosthetics that adapt to user movement
Amyotrophic Lateral Sclerosis (ALS) [34,45,46,47,66]	Implantable brain stimulation devices from NeuroPace	Deep brain stimulation (DBS) surgery for device placement	Improved environmental interaction and device control via neural signals
Cerebral Palsy [34,40,41,42]	AI-powered prosthetics from Medtronic for tailored mobility solutions	Surgical implantation of neurostimulators to support motor control	Increased task performance and greater autonomy
Amputations [34,35,36,67,68,69]	Intelligent prosthetics like Össur’s Proprio system, responsive to terrain	Amputation surgery followed by prosthetic fitting	More precise motor functions and more natural gait simulation

**Table 6:** Health challenges in bone regeneration that AI can resolve.

Bone Regeneration Challenge	Technology	Surgical Technique	Benefit
Complex Fractures [34,57,58,70]	Predictive surgical planning models by Orthofix	Osteosynthesis using bone stimulators or AI-guided implant designs	Faster recovery through personalized treatment and enhanced fracture healing
Advanced Osteoporosis [34,61,62,63,71]	AI-driven, 3D-printed implants by Bioretec	Implant-based fracture fixation or joint replacement (osteosynthesis or arthroplasty)	Improved bone strength and reduced risk of recurrent fractures with biologically compatible implants
Bone Tumors [34,49,50,72,73]	Regenerative biomaterials and AI-assisted grafts by Xtant Medical	Oncologic surgery for bone tumor resection followed by AI-guided bone graft placement	Accurate anatomical post-tumor resection with enhanced structural integrity
Congenital Deformities [34,59,60,74]	AI-customized prosthetics	Corrective osteotomies with patient-specific prosthesis placement	Functional and aesthetic improvements via individualized surgical correction
Traumatic Injuries [34,51,52,53,54,55,56]	Bone stimulation and cell therapy from Bioventus	Surgical repair with bone stimulators and regenerative materials (osteosynthesis)	Effective regeneration of damaged bone using advanced biomaterials and stimulation
